# Comparison of elasticity changes in the paraspinal muscles of adolescent patients with scoliosis treated with surgery and bracing

**DOI:** 10.1038/s41598-024-56189-w

**Published:** 2024-03-07

**Authors:** Hyun Ji Lim, Haesung Yoon, Jisoo Kim, Kyunghwa Han, Yuri So, Mincheol Park, Kun-Bo Park, Mi-Jung Lee

**Affiliations:** 1 Department of Pediatric Radiology, Severance Children’s Hospital, Yonsei University College of Medicine, 50-1 Yonsei-Ro, Seodaemun-Gu, Seoul, 03722 Republic of Korea; 2 Department of Radiology, Research Institute of Radiological Science and Center for Clinical Imaging Data Science, Yonsei University College of Medicine, Seoul, South Korea; 3 Department of Biostatistics and Computing, Yonsei University Graduate School, Seoul, South Korea; 4 Division of Pediatric Orthopedic Surgery, Severance Children’s Hospital, Yonsei University College of Medicine, Seoul, South Korea; 5 Department of Radiology and Research Institute of Radiological Science, Severance Hospital, Yonsei University College of Medicine, 50-1 Yonsei-Ro, Seodaemun-Gu, Seoul, 03722 Republic of Korea

**Keywords:** Biophysics, Diseases, Medical research

## Abstract

Scoliosis is a three-dimensional spinal deformity, and paraspinal muscles play an important role as stabilizers of the spinal curve. In this prospective study, we compared elasticity changes in the paraspinal muscles of adolescent patients with scoliosis after surgery or bracing. Elasticity was measured on the concave and convex sides of the paraspinal muscles at the apex of the curve at the beginning of treatment and 6 and 12 months after treatment. Twenty-six patients with correction surgery (n = 15) or bracing (n = 11) were included. At initial evaluation, the Cobb angle was larger in the surgery group (72.3 ± 20.2° in surgery vs. 30.6 ± 5.1° in brace, p < 0.001). The estimated mean elasticity value of the paraspinal muscles was lower in the surgery group at baseline on the convex side (15.8 vs. 22.8 kPa, p = 0.037) and 6 months on both the concave (12.1 vs. 22.7 kPa, p = 0.004) and convex (13.4 vs. 23.8 kPa, p = 0.005) sides. There was a significant stiffness decrease from baseline to 6 months on the concave side in the surgery group (5.9 kPa, p = 0.025). However, the elasticity change recovered at 12 months without significant differences between the two groups.

## Introduction

Scoliosis is a three-dimensional spinal deformity characterized by a lateral curvature of at least 10°. Scoliosis requires closer attention in adolescence because it is a period of growth. Adolescent idiopathic scoliosis (AIS) without underlying cause is the most common type of scoliosis with global prevalence ranging from 0.5 to 12%, although percentages vary according to geography, patient sex and age^1^. Other than AIS, scoliosis can be a consequence of neuromuscular disease or congenital scoliosis from heterotopic vertebrae and ribs^2^. For any cause of scoliosis, the asymmetric loading that defines the deformity is thought to begin in the paraspinal muscles^3,4^.

The treatment of scoliosis at an adolescent age depends on the severity of the curvature or growth potential remaining and involves bracing and surgery. Patients with spinal curvature of 25­40° with one to two years or more of remaining growth can undergo bracing^5^. If the curve progresses or is severe, operative treatment with instrumentation is recommended to realign and stabilize the affected portion of the spine.

The paraspinal muscles play a pivotal role in the symmetric growth, stabilization, and development of the spine^6^. Previous studies have reported asymmetry and imbalances in the paraspinal muscles as a cause of scoliosis^7,8^. Specifically, there is an asymmetrical distribution of muscle volume, with a larger volume observed on the convex side while fatty deposition and muscle atrophy are detected on the concave side at the apex spinal level^9^. Asymmetry in the volume and composition of paraspinal muscles may be linked to compressive force caused by spinal curvature^10^. However, few publications have noted the post-treatment changes observed in paraspinal muscles. While concerns exist about muscle weakening during bracing, there is only limited research available regarding how these muscles change after treatment^11^. Also, despite it being known that bony fusion typically occurs within 6 months after surgery, we still do not know when these muscles recover^12^.

Ultrasound shear wave elastography (SWE) is a fast and safe technique utilized to assess tissue stiffness in various clinical conditions, such as liver fibrosis, by measuring the speed of shear wave propagation in tissues^13^. Numerous studies have explored the application of SWE to assess muscle properties, with the expectation that alterations in muscle structure and composition, including changes in fat and collagen, can lead to changes in stiffness^14–16^. Traditional methods for assessing muscle properties include procedures like biopsy or MRI, but SWE provides a simpler and safer method of assessment^17,18^. Attempts have been made to evaluate muscle properties using SWE for different muscles, such as the biceps brachii and biceps femoris, across various conditions like cerebral palsy^19–21^. While some studies have reported on the stiffness of paraspinal muscles in scoliosis patients, none has yet to compare differences according to treatment methods^22–24^.

Therefore, the purpose of this study was to evaluate changes in muscle elasticity using ultrasound SWE in adolescents with scoliosis. We compared elasticity changes between patients treated with surgery and bracing until 12 months after treatment.

## Results

### Clinical data

A total of 44 scoliosis patients were recruited during the study period and 18 patients were excluded due to surgery with growing rods (n = 3) or bracing for less than 10 h (n = 15). Finally, 26 scoliosis patients (M: F = 4: 22, mean age 13.7 ± 2.0 years) with surgery (n = 15) or brace (n = 11) treatment were analyzed. The wearing time of brace was 12–16 h in the included patients. The surgery group was of older age (14.7 ± 1.9 vs. 12.4 ± 1.1 years, p = 0.002). More females had scoliosis, in both the surgery and brace groups (73.3% [11/15] vs. 100% [11/11], p = 0.113). There was no significant difference in body mass index between the surgery and brace groups (20.1 ± 5.9 vs. 17.8 ± 1.3 kg/m^2^, p = 0.150). In the surgery group, eight patients had underlying diseases, including neuromuscular disease (n = 7) and neurofibromatosis (n = 1), while the other seven patients had AIS. All patients in the brace group had AIS (n = 11). In the surgery group, multi-level correction surgery was performed, ranging from T2 to S1. The lower instrumented vertebra was over T12 in all patients. There was no patient lost to follow-up during this study.

The mean baseline Cobb angle was significantly different between the surgery and brace groups (72.3 ± 20.2° vs. 30.6 ± 5.1°, p < 0.001). The post-treatment Cobb angle, which was measured immediately after treatment, was also significantly different between the surgery and brace groups (26.9 ± 15.6° vs. 14.5 ± 5.6°, p = 0.009) (Table 1).

**Table 1:** Clinical information and paraspinal muscle elasticity values of the surgery and brace groups.

			Surgery (n = 15)	Brace (n = 11)	P-value
Age (years)			14.7 ± 1.9	12.4 ± 1.1	**0.002**
Sex (male:female)			4:11	0:11	0.113
Mean baseline Cobb angle (°)			72.3 ± 20.2	30.6 ± 5.1	** < 0.001**
Post-treatment Cobb angle (°)			26.9 ± 15.6	14.5 ± 5.6	**0.009**
Paraspinal muscle elasticity (kPa)	At baseline	Concave side	18.0 (13.7–22.3)	21.1 (16.1–26.1)	0.346
		Convex side	15.8 (11.6–20.1)	22.8 (17.8–27.8)	**0.037**
	At 6 months after treatment	Concave side	12.1 (7.3–16.9)	22.7 (17.3–28.0)	**0.004**
		Convex side	13.4 (8.6–18.1)	23.8 (18.4–29.1)	**0.005**
	At 12 months after treatment	Concave side	15.6 (11.0–20.2)	17.7 (12.1–23.3)	0.567
		Convex side	16.8 (12.2–21.4)	19.9 (14.3–25.4)	0.401

Significant values are in bold.

Data are means ± standard deviations or estimated means (95% confidence interval [CI]).

Post-treatment Cobb angle was measured 12 months after treatment.

### Comparison of paraspinal muscle elasticity between the surgery and brace groups

Individual differences in paraspinal muscle elasticity were observed. A linear mixed model was used to estimate the mean elasticity values on the concave and convex sides. In the surgery group, the estimated mean elasticity on the concave side was 18.0 kPa (95% CI 13.7–22.3) at baseline, 12.1 kPa (95% CI 7.3–16.9) at 6 months, and 15.6 kPa (95% CI 11.0–20.2) at 12 months. The estimated mean elasticity on the convex side was 15.8 kPa (95% CI 11.6–20.1) at baseline, 13.4 kPa (95% CI 8.6–18.1) at 6 months, and 16.8 kPa (95% CI 12.2–21.4) at 12 months. The estimated mean elasticities of the brace group are presented in Table 1.

**Figure 1:**
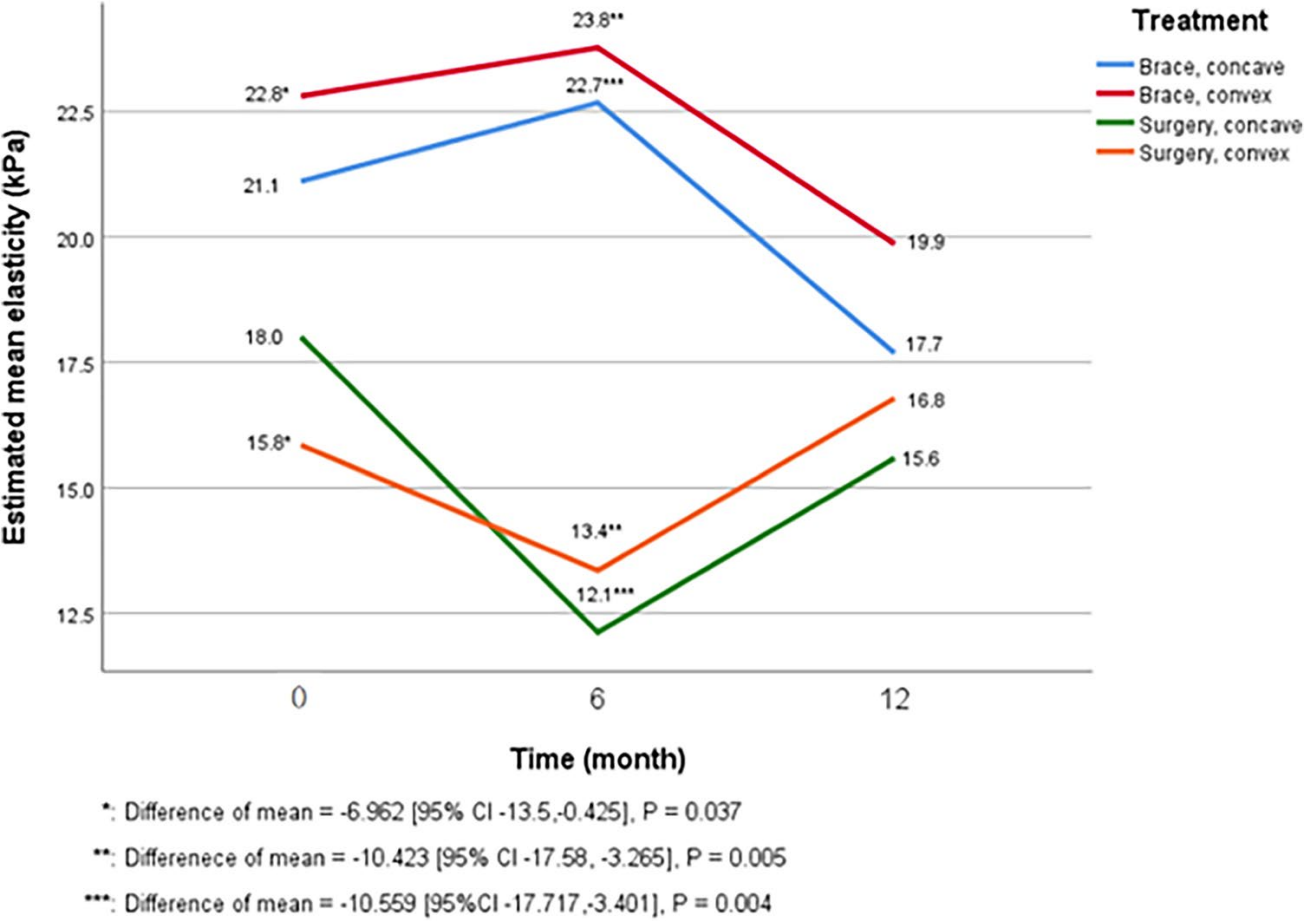
Changes in the estimated mean elasticity values of the paraspinal muscles in the surgery and brace groups. The graph shows the estimated mean elasticity values of the paraspinal muscles at each time point. The surgery group shows lower muscle elasticity values at all time points than the brace group. The difference was statistically significant at baseline on the convex side (15.8 vs. 22.8 kPa, p = 0.037*) and at 6 months on both the convex (13.4 vs. 23.8 kPa, p = 0.005**) and concave (12.1 vs. 22.7 kPa, p = 0.004***) sides. There was a significant stiffness decrease from baseline to 6 months on the concave side in the surgery group (5.9 kPa, p = 0.025). There were no significant differences in elasticity between baseline and 12 months in both groups.

The surgery group consistently exhibited lower estimated mean elasticity compared to the brace group across all time points (Fig. 1). Significant differences in paraspinal muscle elasticity were observed between the two groups at baseline on the convex side (15.8 vs. 22.8 kPa, p = 0.037) and at 6 months on both the concave (12.1 vs. 22.7 kPa, p = 0.004) and convex (13.4 vs. 23.8 kPa, p = 0.005) sides (Table 1). However, there was no significant difference between the groups at 12 months.

When considering underlying disease in the surgery group, the estimated mean elasticity at baseline in patients with disease was 12.6 kPa (95% CI 7.2–18.1) on the concave side and 17.8 kPa (95% CI 12.3–23.3) on the convex side compared with that in patients without underlying disease as 19.5 kPa (95% CI 13.7–25.4) on the concave side and 18.2 kPa (95% CI 12.4–24.1) on the convex side. There was no statistically significant difference between the patients with and without underlying disease in the surgery group (p = 0.091 on concave side and p = 0.910 on convex side).

### Comparison of elasticity change over time in paraspinal muscles between the surgery and brace groups

When considering the temporal change of paraspinal muscle elasticity in each group, both groups demonstrated temporal changes in elasticity (Fig. 1). In the surgery group, elasticity decreased at 6 months and recovered at 12 months. During 12 months, elasticity decreased significantly on the concave side from baseline to 6 months in the surgery group (estimated mean elasticity change 5.9 kPa [95% CI 0.7–11.0], p = 0.025) (Table 2). However, in the brace group, there were no significant changes in elasticity for 12 months. The paraspinal muscle elasticity was not significantly different between baseline and 12 months after treatment in both groups (Table 2). Furthermore, no asymmetry in elasticity was observed between the concave and convex sides of the paraspinal muscles during the study period (Table 3).

**Table 2:** Comparison of temporal changes in the paraspinal muscle elasticity of the surgery and brace groups.

Treatment groups	Measurement side	Time points being compared		Paraspinal muscle elasticity change (kPa)	P-value
Surgery	Concave	Baseline	6 months	5.9 (0.7–11.0)	**0.025**
		6 months	12 months	− 3.5 (− 8.8–1.9)	0.204
		Baseline	12 months	2.4 (− 2.6–7.4)	0.340
	Convex	Baseline	6 months	2.5 (− 2.6–7.6)	0.338
		6 months	12 months	− 3.4 (− 8.8–2.0)	0.209
		Baseline	12 months	− 0.9 (− 6.0–4.1)	0.712
Brace	Concave	Baseline	6 months	− 1.6 (− 7.3–4.2)	0.592
		6 months	12 months	5.0 (− 1.4–11.4)	0.123
		Baseline	12 months	3.4 (− 2.6–9.4)	0.260
	Convex	Baseline	6 months	− 1.0 (− 6.7–4.8)	0.741
		6 months	12 months	3.9 (− 2.4–10.3)	0.225
		Baseline	12 months	3.0 (− 3.0–9.0)	0.331

Significant values are in bold.

Data are estimated means (95% confidence intervals [CI]).

**Table 3:** Comparison of paraspinal muscle elasticity between the concave and convex sides.

Treatment group	Measurement time	Paraspinal muscle elasticity difference between the concave and convex side (kPa)	P-value
Surgery	At baseline	2.2 (− 2.5–6.8)	0.360
	At 6 months	− 1.2 (− 6.7–4.2)	0.652
	At 12 months	− 1.2 (− 6.4–4.0)	0.650
Brace	At baseline	− 1.7 (− 7.1–3.7)	0.536
	At 6 months	− 1.1 (− 7.1–4.9)	0.717
	At 12 months	− 2.2 (− 8.5–4.2)	0.499

Data are estimated means (95% confidence intervals [CI]).

## Discussion

Scoliosis is a disease that progresses with growth and there are diverse causes of scoliosis. Whatever the reason, as scoliosis progresses, the muscles located at the concave and convex sides also change, and these muscle changes can further affect scoliosis^4,7,8^. However, there have been few studies on the temporal change of paraspinal muscles in scoliosis according to the method of treatment. As scoliosis is a three-dimensional disease affected by asymmetric muscles or sarcopenia^8,25^, muscle status including stiffness will change during bracing or surgical treatment. Our study is the first to report on the changes that occur in muscle elasticity during treatment using SWE in scoliosis patients of adolescent age. Muscle stiffness significantly changed during the first 6 months in patients who received surgery and returned to pretreatment values at 12 months after treatment. However, there was no significant change in the muscle stiffness of patients who received braces for 12 months. These results suggest that elasticity measurements can show physical or mechanical changes in the paraspinal muscles quantitatively during treatment.

The treatment of scoliosis in adolescents consists of bracing or surgery and its direction depends on the severity of the curvature^5^. Both treatments decrease the degrees of scoliosis, although the purpose of bracing is to slow down its progression^1^. While limited, previous studies have explored changes in paraspinal muscles following treatment for scoliosis in this age group. An animal experimental study found that six months after spinal fusion, paraspinal muscles showed atrophy with hypertrophy of the adjacent regions^26^. Another human study also showed a significant decrease in the paraspinal muscle area after surgery with a mean follow-up period of 9.9 years^27^. Muscular activity evaluated with electromyography also showed asymmetry between fused and unfused regions in idiopathic scoliosis patients^28^. In a previous study, after surgery with a mean follow-up period of 2 years, paraspinal muscles in the thoracic region showed lower activity which was probably due to atrophy and those in the lumbar region showed higher muscular activity which was thought due to muscle hypertrophy^28^. However, there was no study about early postoperative changes in paraspinal muscles and a limited study that included patients treated with braces^11^.

Assessing muscle stiffness using SWE can reflect the structure and composition of muscles^29^. Various studies have found stiffness to increase during muscle contraction compared to the relaxed state, and SWE has shown reliable repeatability when environmental factors, such as contraction or relaxation and measurement depth, are appropriately standardized^20,30,31^. Regarding paraspinal muscles, stiffness was measured reliably in the rest position^32^. However, there was a limited study about paraspinal-muscle SWE in younger scoliosis patients^24^.

At baseline, there was no asymmetry of elasticity in paraspinal muscles between the concave and convex sides in both the brace and surgery groups. These findings are consistent with a previous study^24^. When considering the asymmetry of paraspinal muscles including volume between the concave and convex sides in adolescent-age scoliosis^8,25^, this absence of asymmetry in elasticity at baseline might be another characteristic of paraspinal muscles in scoliosis. This is further supported by another study in which patients with idiopathic scoliosis had asymmetric electromyographic activity in the paraspinal muscles, which was diminished by spine fusion^28^. Even though morphologic changes may occur in the paraspinal muscles in scoliosis, the balance of muscle stiffness can be preserved symmetrically. Treatment can change this balance as was seen in our study.

Our study is the first to show changes in muscle elasticity after treatment using SWE in both the surgery and brace groups. The surgery group consistently showed lower estimated mean stiffness compared to the brace group across all time points, albeit not statistically significant for all data. This may reflect the degree of underlying myopathy in the surgery group, which had more severe scoliosis. This is in line with prior studies in which the paraspinal muscles in scoliosis patients of adolescent age had muscle atrophy, alternation in hyaline fibers, increased mitochondrial proliferation, and changes in calmodulin levels^33,34^. These histopathologic changes in muscle tissue could potentially manifest as differences in the tissue elasticity measured by SWE. Our results also suggest significant changes in paraspinal muscle stiffness during the first 6 months of treatment for both surgery and bracing. In the surgery group, there was a significant decrease in elasticity at 6 months, followed by recovery to baseline at 12 months. This decrease in elasticity might be attributed to the surgical intervention itself, potentially from tissue injury during dissection and instrumentation. On the other hand, the brace group showed an increased elasticity at 6 months, which returned to baseline by 12 months, although without statistical significance. This temporary increase in elasticity in the brace group may be a result of the mechanical pressure and stretching applied by the brace, potentially reducing the extent of unused paraspinal muscles^35^. Our study data also showed difference of bilateral paraspinal muscles before and after surgery in the surgery group as more decreased stiffness in the concave side (estimated mean elasticity change 5.9 kPa [95% CI 0.7–11.0], p = 0.025). There could be various interpretations of the change difference in elasticity of bilateral paraspinal muscles before and after surgery in scoliosis. It is thought that the concave side muscles have a strong pull before surgery, and then the stiffness decreases as it is stretched out after surgery.

Our study exhibited some disparities compared to previous research which reported increased elasticity in the paraspinal muscles on both the concave and convex sides 6 months after surgery in ten adolescent scoliosis patients^24^. Differences in the elasticity patterns could be due to variations in the severity of scoliosis, underlying disease, and the limited number of patients. Further studies with a large number of patients are needed to validate true differences in muscle stiffness according to patient condition and treatment options.

There are several limitations in this study. First, the surgery group included patients with underlying neuromuscular diseases, which might have affected the elasticity of their muscles. Second, the patients included in this study exhibited varying degrees of scoliosis that involved different spinal levels. Paraspinal muscle elasticity was not consistently measured at the same level. Third, the study only assessed patients for 12 months, and we could not assess long-term changes and outcomes with this data in this study. Future research should consider conducting long-term analyses to better understand how these changes evolve over time. In addition, we did not collect the data of histological information of the paraspinal muscles. Future studies incorporating histologic information are necessary to accurately understand the characteristics of paraspinal muscles in scoliosis patients.

In conclusion, the elasticity of the paraspinal muscles changed during scoliosis treatment in patients of adolescent age. The surgery group showed decreased muscle stiffness 6 months after surgery, but muscle stiffness returned to pretreatment values at 12 months, compared with the brace group. The detection of these changes could help understand the mechanical properties of paraspinal muscles during treatment in adolescent scoliosis patients who are experiencing growth.

## Methods

### Patients and clinical data

This prospective study was approved by the Institutional Review Board of our institution (IRB No. 1-2020-0038). All studies were conducted per the ethical guidelines of the Declaration of Helsinki. Informed consent was obtained from all participating patients and guardians before examination. Consecutive patients aged 10 to 18 years diagnosed with scoliosis using spine plain film at a tertiary hospital were enrolled between August 2020 and September 2021 and followed until September 2022. The inclusion criteria were: (a) age between 10 and 18 years old to exclude early onset scoliosis^36^, (b) spinal curvature greater than 20° Cobb angle, (c) no history of spinal surgery, and (d) no history of scoliosis treatment. The surgery group included patients who underwent scoliosis correction surgery, excluding growing rod procedures. The TLSO bracing was applied in patients with AIS before menstruation, Cobb angle of 25–40°, and Risser grade 0–1^37^. In the brace group, we recommended wearing the brace for at least 12 h^38^. We excluded patients who wore a brace for less than 10 h. Clinical information, including age, sex, underlying disease, body mass index, and treatment, was collected. Spine deformity data, including the baseline Cobb angle and post-treatment Cobb angle, were obtained.

### Elasticity assessment and image analysis

**Figure 2:**
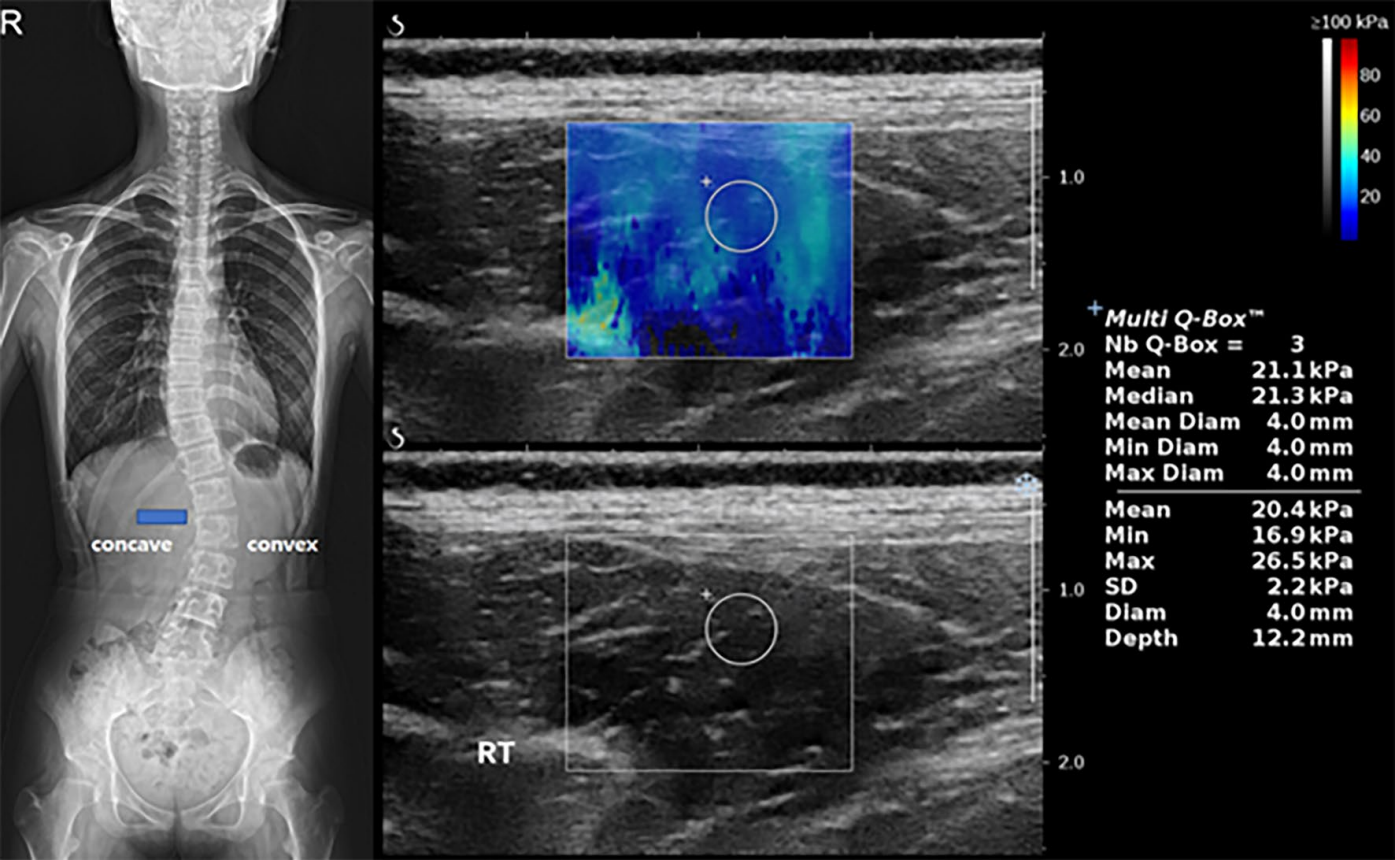
A representative case of shear wave elastography (SWE) measurement in a 12-year-old girl with adolescent idiopathic scoliosis. (**A**) The most curved level was determined using the standing whole spine plain film of the anteroposterior view, and it was L1/2. The blue box shows the location of the ultrasound probe when measuring SWE in the paraspinal muscle of the concave side. (**B**) The SWE image was obtained on the right side (the concave side of this patient) of the paraspinal muscle at the apex of the curve using a high-frequency linear probe. A round region of interest was drawn and the mean value of SWE was obtained (21.1 kPa in this image).

Elasticity was measured at the concave and convex sides of the most curved paraspinal muscles using shear wave elastography at the beginning of treatment, 6 months after treatment, and 12 months after treatment (Fig. 2). Patients were positioned prone with arms placed on the sides of their bodies with their heads in the neutral position and asked to fully relax. Ten regions of interests (ROIs) were marked at the paraspinal muscle lateral to the spinous process at the most curved level after referring to the spine plain film. Four pediatric radiologists under the supervision of one experienced radiologist (M.J.L, 21 years of experience) measured elasticity under the same conditions using the same sonography machine (Aixplorer, SuperSonic Imagine, Aix-en-Provence, France). A high-frequency linear probe (SL10-2) was used to measure elasticity at 1–3 cm depth with an ROI 4 mm in diameter in the paraspinal muscles. The mean elasticity was defined as the mean value of the ten ROIs at each side of the spine.

### Statistical analysis

Statistical analysis was performed by a biostatistician (K. H., with 16 years of experience) using SPSS software version 25.0 (IBM Corp., Armonk, NY, USA) and R software version 4.1.1 (https://www.R-project.org/, Vienna, Austria). The normal distribution of the data was confirmed by the Shapiro–Wilk test. To compare the clinical and radiologic features of the surgery and brace groups, the t-test was used for continuous variables, while the chi-square test was used for categorical variables. The linear mixed model was used to analyze the stiffness of the paraspinal muscles between the surgery and brace groups according to time. Using this model, we considered repeated measurements by incorporating patients as random effects. The fixed effects included treatment (bracing or surgery), time (0, 6, and 12 months), and measurement side (concave or convex). The model incorporated interactions between two factors and three-way interactions between three factors in terms of treatment, time, and measurement side. Means and 95% confidence intervals (CIs) were estimated based on the model with the least square method. P values less than 0.05 were considered statistically significant.

## Data Availability

The datasets generated during and/or analyzed during the current study are not publicly available due to individual privacy could be compromised but are available from the corresponding author on reasonable request. The de-identified participant data will be shared. The patient’s data regarding age, Cobb angle, muscle elasticity can be shared. All the other study protocols were already shared in this manuscript. Data can be provided within one month from the time of request. Additionally, data is only available for review purposes for one month. The access criteria data will be shared to only the editors for review purpose only.

## Abbreviations

AIS: Adolescent idiopathic scoliosis
ROI: Region of interest
SWE: Shear wave elastography
CI: Confidence interval
